# Patterns of myelinated nerve fibers loss in transthyretin amyloid polyneuropathy and mimics

**DOI:** 10.1002/acn3.51599

**Published:** 2022-06-04

**Authors:** Kang Du, Xujun Chu, Yuwei Tang, Xutong Zhao, Meng Yu, Yiming Zheng, Jianwen Deng, He Lv, Wei Zhang, Zhaoxia Wang, Yun Yuan, Lingchao Meng

**Affiliations:** ^1^ Department of Neurology Peking University First Hospital 8 Xishiku Street, Xicheng District Beijing 100034 China; ^2^ Beijing Key Laboratory of Neurovascular Disease Discovery Beijing 100034 China

## Abstract

**Objective:**

The present study was intended to analyze the characteristics of myelinated nerve fibers density (MFD) of transthyretin amyloid polyneuropathy (ATTR‐PN) and other similar neuropathies.

**Methods:**

A total of 41 patients with ATTR‐PN, 58 patients of other common peripheral neuropathies, and 17 age‐and gender‐matched controls who visited the First Hospital of Peking University and performed sural nerve biopsy between June 2007 and August 2021 were included for analysis of MFD.

**Results:**

Except the vasculitic neuropathy group, the total and small MFD of patients in the ATTR‐PN group were significantly lower than those of other disease groups. There was an obvious negative correlation between the total MFD and the disease course in the ATTR‐PN group. The disease course of early‐onset and late‐onset symptoms was similar, but the loss of large myelinated nerve fibers (MF) was more severe for the latter. In addition, all late‐onset and most early‐onset patients had severely reduced MFD after a 2 years' disease course. The MFD in ATTR‐PN patients was negatively correlated with Neuropathy Impairment Score (NIS) and Norfolk Quality of life‐diabetic neuropathy (Norfolk QOL‐DN) score.

**Conclusion:**

MF is lost differently in ATTR‐PN and in other common peripheral neuropathies. The late‐onset and early‐onset ATTR‐PN patients have different patterns of loss of large and small MF.

## Introduction

Transthyretin amyloid polyneuropathy (ATTR‐PN) is a progressive and life‐threatening disease caused by *TTR* gene mutation with an autosomal dominant inheritance pattern. The disease is characterized by transthyretin (TTR) amyloid fibrils deposition in multiple organs, mostly leading to sensory‐motor neuropathy, autonomic dysfunction, and cardiomyopathy, accompanied by involvement of other organs.^1^ According to the age of onset, ATTR‐PN is classified as early‐onset before 50 years old and late‐onset after 50 years old. Progressive polyneuropathy and cardiomyopathy are more common in late‐onset patients, while progressive polyneuropathy with prominent autonomic dysfunction is common in early‐onset ones.^1^ Electrophysiological studies are generally compatible with sensory‐motor axonal polyneuropathy, but sometimes with demyelinating features.^2^ Pathologically, the loss of unmyelinated nerve fibers occurs early, reduced densities of small and then larger myelinated nerve fibers are observed, and blood vessels are frequently invaded and destroyed by TTR accumulation.^3^, ^4^ Diagnosis of ATTR‐PN mainly depends on amyloid deposits in the tissues and *TTR* gene testing. So far, more than 150 mutations have been described in the *TTR* gene.^5^ The prevalence of ATTR‐PN with different mutations varies according to ethnicity and geographic locations. *TTR* Val30Met mutation is the most common pathogenic variant overall.^5^

Late‐onset and sporadic ATTR‐PN cases, which were found to be common in mainland China in our previous study, are prone to misdiagnosis.^6^, ^7^ The common misdiagnosed diseases include chronic inflammatory demyelinating polyradiculoneuropathy (CIDP), chronic idiopathic axonal polyneuropathy (CIAP), polyneuropathy, organomegaly, endocrinopathy, monoclonal gammopathy, and skin change (POEMS) syndrome, late‐onset Charcot–Marie–Tooth type 2 (LO‐CMT2) and other axonal or mixed neuropathy.^6^

Sural nerve biopsy is preferred when clinicians are confused about the diagnosis that is based on clinical and electrophysiological findings alone.^8^ However, the Congo red staining is not always highly sensitive as was proved by our experience and previous studies.^4^, ^7^ The sensitivity in different studies, ranges from 40% to 80%.^4^, ^7^ Negative Congo red staining in the tissue in ATTR‐PN is a common cause of diagnostic delay and loss of the optimal chance of treatment.^9^ The number of myelinated nerve fibers are intuitive to observe, and it is one of the most important indicators of disease progression. Therefore, to prevent misdiagnosis of ATTR‐PN patients, we should attach importance to not only Congo red staining, but also to the extent and speed of loss of myelinated nerve fibers. In this study, we investigated the loss pattern of myelinated nerve fibers of ATTR‐PN and other common peripheral neuropathies.

## Materials and Methods

### Subjects

Between June 2007 and August 2021, we retrospectively analyzed the sural nerve pathology of 41 patients (thirty males and eleven females) with ATTR‐PN, 10 patients (three males and seven females) with CIDP, 14 patients (seven males and seven females) with CIAP, 6 patients (five males and one female) with LO‐CMT2, 8 patients (two males and six females) with POEMS syndrome, 13 patients (seven males and six females) with definite vasculitic neuropathies, 7 patients (three males and four females) with toxic neuropathy, and 17 controls (eleven males and six females), who were diagnosed in Peking University First Hospital. All ATTR‐PN patients were diagnosed according to the diagnostic criteria.^10^

For the diagnosis of definite CIDP, the diagnostic criteria proposed by the Joint Task Force of the European Federation of Neurological Societies and the Peripheral Nerve Society (EFNS/PNS) were adopted.^11^ All the patients had evidence of demyelination with negative Congo red staining in sural nerve biopsy. In addition, they all got improved after immunotherapy.

The diagnosis of CIAP was based on previously described criteria.^12^, ^13^ All patients had an insidious onset and slow or no progression for at least 6 months. And all patients had a distribution of sensory or sensorimotor symptoms and signs compatible with axonal polyneuropathy in electrophysiology. In addition, they were finally diagnosed as CIAP in the absence of any possible cause of neuropathy by extensive clinical and laboratory examinations, including glucose tolerance test, serum vitamin B12 levels, homocysteine, infection screening, autoimmune‐related antibodies, paraneoplastic antibodies, immunofixation electrophoresis of serum and urine, serum free light chain, cerebrospinal fluid examination and so on. All patients were excluded from ATTR‐PN by negative *TTR* gene screening.

All LO‐CMT2 patients were diagnosed by Next Generation Sequencing (NGS) with *MFN2* in three patients, and *MPZ* (relating to CMT2J subtype), *NEFL,* and *GDAP1* in one patient, respectively. All these variations had been reported and were considered to be likely pathogenic or pathogenic variations according to the criteria by the American College of Medical Genetics and Genomics (ACMG).^14^

All POEMS syndrome patients were diagnosed according to the latest criteria of 2019.^15^ The diagnosis of POEMS syndrome was confirmed when both of the mandatory major criteria, one of the three other major criteria, and one of the six minor criteria were present.

All vasculitic neuropathy patients were diagnosed based on sural nerve biopsies, and compatible with diagnostic criteria of pathologically definite vasculitic neuropathy proposed by Peripheral Nerve Society Guideline.^16^

All toxic neuropathy patients had a definite history of toxic exposure. Meanwhile, other common causes of peripheral neuropathy were excluded, such as diabetic neuropathy, alcoholic neuropathy, and paraneoplastic neuropathy. The peripheral neuropathy‐related substances included chemotherapeutic drugs (platinum compounds, taxanes etc.) in four patients, thalidomide in two patients, and organophosphorous pesticide, telbivudine, and acrylamide in one patient, respectively. All these chemotherapies or toxicants related neuropathies had been reported previously.^17^, ^18^

Seventeen sural nerve tissues from subjects who had no definite pathological abnormalities, which were judged by a group of experienced neuropathologists, were selected as pathomorphological normal controls.

### Ethic statement

All the subjects associated with this study underwent sural nerve biopsies and signed informed consent, and this study was approved by the Ethics Committee of First Hospital of Peking University.

### Clinical evaluation of ATTR‐PN

The mean age at which all the ATTR‐PN subjects underwent biopsies was 50.3 ± 13.3 years, with 21 early‐onset patients and 20 late‐onset patients. All patients had *TTR* gene mutation, with Val30Met in thirteen, Val30Leu in four, Ala97Ser in three, Lys35Asn in three, Ala36Pro, Phe33Leu, Phe33Val, Gly47Arg, Val30Ala, Gly83Arg, and Glu61Lys in two, Glu42Gly, Ser77Phe, Thr59Lys, and Val28Ser in one patient, respectively.

All the ATTR‐PN patients were inquired about their disease history and had a focused neurological examination of measurement scales performed, including Coutinho stages, Neuropathy Impairment Score (NIS), Norfolk Quality of life‐diabetic neuropathy (Norfolk QOL‐DN) score, and Composite Autonomic Symptom Score 31 (COMPASS‐31). The onset of disease was defined as the progressive sensory‐motor neuropathy or/and autonomic neuropathy. A disease course of more than 2 years was considered as a long course.

### Sural nerve biopsies

Sural nerve biopsies were performed as described previously.^19^ The morphometric indices were assessed in toluidine blue‐stained semithin sections. Given the variability of different nerve fascicles in each case, we manually counted the total number of myelinated nerve fibers (MF) from at least three fascicles on complete transverse sections of sural nerves on light microscopic images, captured at a magnification of 400× covering the maximum nerve area, in order to evaluate the extent of MF loss in each case objectively. The total, the large(>7 μm), and the small (≤7 μm) myelinated nerve fibers densities (MFD)^3^, ^20^ were calculated by using Photoshop CC 2018 software (Adobe Systems, San Jose, CA). The density of unmyelinated fibers was not assessed. The mean small, large, and total MFD of the control group were used for calculating the MF loss per year of ATTR‐PN, CIAP, and CIDP groups, that is, the MF loss per year = [Mean MFD (Controls) ‐ Mean MFD (Patients)]/Disease course. The total MFD with less than 1000/mm^2^ was considered as severe MF loss.

### Statistical analysis

For statistical analysis, IBM SPSS Statistics, version 26, was used. As most of the disease and control groups showed a non‐normal distribution (as evaluated by single sample K‐S test), Pearson's chi‐square test or Fisher's exact test, Mann–Whitney test and Kruskal–Wallis test were used to study the differences between the ATTR‐PN group, non‐ATTR‐PN groups, and the control group. Receiver operating characteristic (ROC) curve analysis was performed to evaluate the use of MF loss per year in differentiating ATTR‐PN from CIDP and CIAP groups. The area under the curve (AUC) was calculated. The value of the Youden index at its maximum was taken as the cut point for the diagnosis of ATTR‐PN, and the sensitivity and specificity were calculated. Two‐sided *p* values were calculated for all analyses, and *p* values <0.05 were considered statistically significant. Spearman's correlation analysis was used to test the correlations between MFD and data of clinical scores.

## Results

### Comparison of MFD between ATTR‐PN and non‐ATTR‐PN group

**Figure 1:**
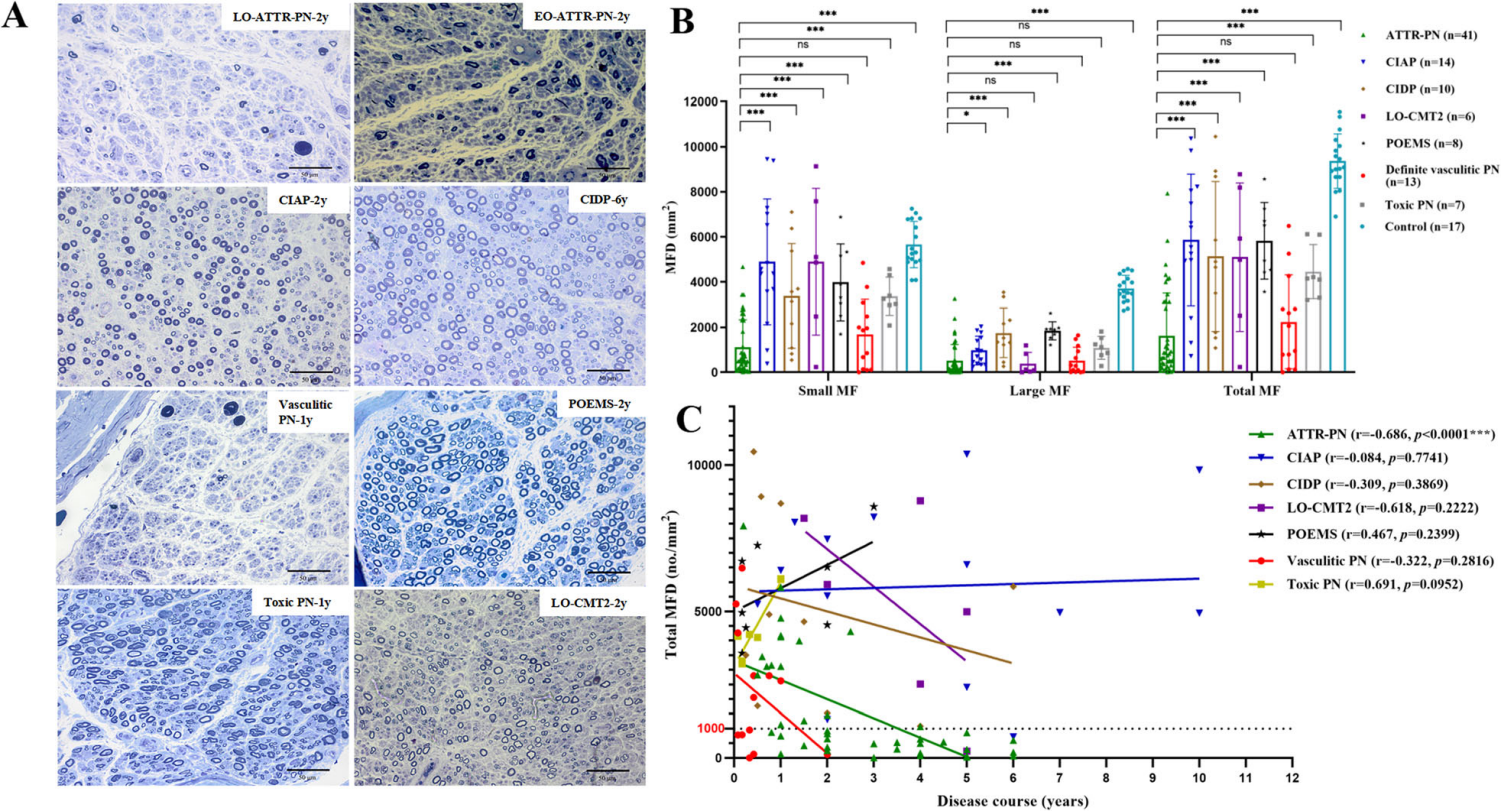
Difference o MFD between ATTR‐PN and other common peripheral neuropathies. (A) The pathological pictures of sural nerves of the typical patients with different disease courses were showed with toluidine blue staining. Patients of ATTR‐PN and vasculitic neuropathy had more severe MFD reductions compared with other disease groups, and the latter had a more rapid disease course. The MFD reduction was more severe in late‐onset than early‐onset patient of ATTR‐PN. (B) A quantitative comparison of small, large, and total MFD between the ATTR‐PN group and other different disease groups. (C) Correlation analysis between the course of disease and total MFD in different disease groups. ATTR‐PN, transthyretin amyloid polyneuropathy; CIAP, Chronic idiopathic axonal polyneuropathy; CIDP, chronic inflammatory demyelinating polyradiculoneuropathy; CMT2, Charcot–Marie–Tooth type 2; EO, early onset; LO, late onset; m, months; MF, myelinated nerve fibers; MFD, myelinated nerve fibers density; PN, polyneuropathy; POEMS, polyneuropathy, organomegaly, endocrinopathy, monoclonal gammopathy, and skin changes; y, years. Scale bars = 50 μm. *, ***Significant difference/correlation at 0.05 and 0.001 levels, respectively.

The clinical features and MFD of all groups were shown in Table 1. The reduction of the total MFD was more prominent in the ATTR‐PN group compared with other disease groups (all *p* < 0.05), as was shown in Figure 1A,B, except the vasculitic neuropathy group(*p* > 0.05). The large MFD of ATTR‐PN, LO‐CMT2, vasculitic neuropathy, and toxic neuropathy groups were all significantly reduced, but there was no statistically significant difference between ATTR‐PN and LO‐CMT2, vasculitic neuropathy or toxic neuropathy group (all *p* > 0.05). As for small MFD, severe reduction was observed in both the ATTR‐PN and vasculitic neuropathy groups, but without significant difference (*p* > 0.05). More significant loss occurred in the ATTR‐PN group than in other disease groups (all *p* < 0.05). Furthermore, the correlations between MFD and the disease course were analyzed. Just as Figure 1C showed, there was a significant negative correlation between the total MFD and the course of disease in the ATTR‐PN group (r = −0.686, *p* < 0.0001). It seemd that there was a more severe loss of the MF but a shorter course in the vasculitic neuropathy group than in other groups. However, the correlations between MFD and the disease courses in these groups were not statistically significant (all *p* > 0.05) except the ATTR‐PN group.

**Table 1:** Clinical features and MFD of each group.

Characteristics	ATTR‐PN	CIAP	CIDP	LO‐CMT2	POEMS syndrome	Vasculitic PN	Toxic PN	Control
	*n* = 41	*n* = 14	*n* = 10	*n* = 6	*n* = 8	*n* = 13	*n* = 7	*n* = 17
Age of biopsy, years, mean (SD)	50.6 (13.0)	60.1 (8.3)	45.9 (14.8)	37.8 (10.0)	46.8 (9.0)	61.8 (13.6)	52.4 (18.6)	45.1 (12.8)
Male, n (%)	30 (73.1)	7 (50.0)	3 (30.0)	5 (83.3)	2 (25.0)	7 (54.0)	3 (42.9)	11 (64.7)
Duration from initial symptom to sural nerve biopsy, y, median (IQR)	2.0 (1.0, 4.0)	4.0 (1.8, 6.3)	0.9 (0.5, 2.5)	4.0 (1.9, 5.0)	0.4 (0.2, 2.0)	0.3 (0.1, 0.6)	0.3 (0.2, 1.0)	NA
Symptoms, n (%)								
SMPN	34 (82.9)	7 (50.0)	9 (90.0)	6 (100.0)	6 (75.0)	11 (84.6)	5 (71.4)	NA
Sensory predominance	6 (14.6)	7 (50.0)	1 (10.0)	0 (0)	2 (25.0)	2 (15.4)	2 (28.6)	NA
Dysautonomia	39 (95.1)	3 (21.4)	3 (30.0)	0 (0)	0 (0)	0 (0)	0 (0)	NA
MFD, mean (SD)								
Total MFD, no./mm^2^	1617.2 (1893.5)	5863.0 (2923.9)	5135.0 (3322.9)	5104.2 (3289.9)	5821.6 (1701.8)	2235.8 (2079.0)	4456.8 (1199.2)	9363.3 (1209.8)
Small MFD, no./mm^2^	1109.9 (1214.4)	4896.4 (2784.2)	3386.1 (2316.9)	4899.2 (3250.5)	3987.8 (1702.6)	1681.6 (1556.1)	3370.1 (841.4)	5656.5 (1026.9)
Large MFD, no./mm^2^	507.4 (736.4)	968.6 (633.9)	1748.9 (1097.9)	378.2 (504.0)	1845.7 (402.8)	518.0 (596.9)	1087.6 (509.5)	3712.0 (585.7)
Small MF/Large MF ratio, mean (SD)	3.9 (3.8)	7.4 (6.8)	2.2 (1.0)	61.3 (60.9)	2.3 (1.2)	5.7 (3.7)	4.2 (3.3)	1.6 (0.4)

Abbreviations: IQR, interquartile range; MF, myelinated nerve fibers; MFD, myelinated nerve fibers density; NA, not available; SD, standard deviation; SMPN, sensory‐motor polyneuropathy.

Small MF/Large MF ratio was not available in five cases of ATTR‐PN, one case of LO‐CMT2, and one case of toxic PN group for severely loss of large MF (large MFD = 0/mm^2^).

### ROC curves of MFD for distinguishing ATTR‐PN from CIAP and CIDP

**Figure 2:**
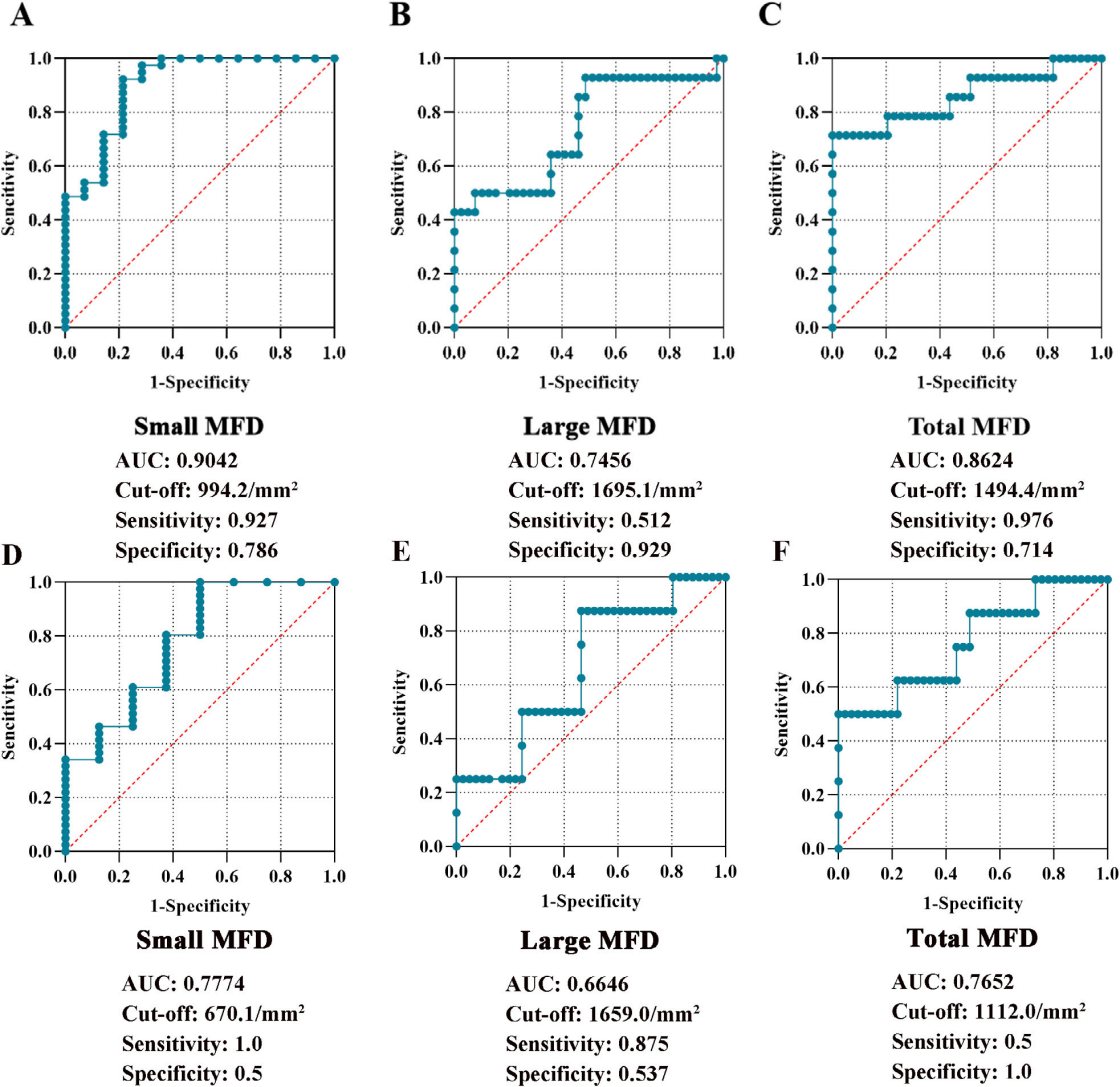
Receiver operating characteristic analysis of MFD in ATTR‐PN, CIAP and CIDP. The degree of MFD loss within a unit time (1 year) for identifying ATTR‐PN from CIAP had a higher sensitivity and specificity in small (A) and total MFD (C), there was a higher specificity but relatively lower sensitivity in that large MFD (B). For ATTR‐PN and CIDP, there was a higher but relatively lower  in both small (D) and large MFD (E), while the result was opposite in total MFD (F). AUC, area under the curve; ATTR‐PN, transthyretin amyloid polyneuropathy; CIAP, Chronic idiopathic axonal polyneuropathy; CIDP, Chronic inflammatory demyelinating polyradiculoneuropathy; MFD, myelinated nerve fibers density.

Based on the results in Figure 1, the ROC curves of the MF loss per year were drawn to compare ATTR‐PN group and the groups of CIAP and CIDP. The cutoff values of the MFD reduction per year were 994.2/mm^2^, 1695.1/mm^2^, and 1494.4/mm^2^ for small, large and total MFD, respectively. The AUCs of the above MFD were 0.9042, 0.7456, and 0.8624, with a high sensitivity (0.927; 0.512; 0.976) and specificity (0.786; 0.929; 0.714), respectively. For ATTR‐PN and CIDP, the cutoff values of the MFD reduction per year were 670.1/mm^2^, 1659.0/mm^2^, and 1112.0/mm^2^ for small, large, and total MFD, respectively. The AUCs of the related MFD were 0.7774, 0.6646, and 0.7652, the sensitivities were 1.0, 0.875, and 0.5, respectively, and the specificities were 0.5, 0.537, and 1.0, respectively (Fig. 2).

### Pathological analysis of sural nerves of ATTR‐PN

#### *Congo red staining of* *ATTR‐PN*

The Congo red positive proportion of nerve biopsies was 17/41 (41.4%) in all ATTR‐PN patients. The early‐onset patients had a higher Congo red positive proportion than  late‐onset ones (52.3% vs. 30.0%), but without significant difference (*p* = 0.2082). Compared with the patients with a short disease duration (≤2 years), the proportion of positive Congo red staining was higher in patients with a long disease duration (>2 years) (61.1% vs. 26.0%), with significant difference (*p* = 0.0308). Considering both the disease duration and age of onset, late‐onset patients with a short disease duration had a lower positive Congo red proportion than early‐onset patients with a long disease duration(16.6% vs. 70.0%), with significant difference(*p* = 0.0274) (Table 2).

**Table 2:** Clinical and pathologic findings of subgroups in ATTR‐PN.

	Early onset (*n* = 21)	Late onset (*n* = 20)	*p* value	Val30Met (*n* = 13)	Non‐Val30Met (*n* = 28)	*p* value
Male, *n* (%)	16 (76.2)	14 (70.0)	>0.7337	9 (69.2)	21 (75.0)	0.7186
Age at onset, y, mean (SD)	37.3 (8.4)	58.7 (4.8)	<0.0001***	58.1 (6.7)	43.0 (12.1)	0.0001***
Positive family History, *n* (%)	19 (90.4)	9 (45.0)	0.0025**	3 (23.0)	25 (89.2)	0.0001***
Initial manifestation						
SMPN, *n* (%)	10 (47.6)	18 (90.0)	0.0063**	11 (84.6)	18 (64.3)	0.2756
AN, *n* (%)	7 (33.3)	2 (10.0)	0.1300	2 (15.4)	6 (21.4)	>0.9999
Others, *n* (%)	4 (19.1)	0 (0)	0.1069	0 (0)	4 (14.3)	0.2883
Disease duration at biopsy, y, IQR	2.0 (1.0, 4.0)	2.0 (1.0, 4.0)	0.8608	1.5 (0.9, 4.0)	2.3 (1.0, 4.0)	0.4142
Positive Congo red staining, *n* (%)	11 (52.3)	6 (30.0)	0.2082	3 (23.0)	14 (50.0)	0.1734
Long course (>2 years), *n* (%)	7 (70.0)	4 (50.0)	0.6305	2 (50.0)	9 (64.2)	>0.9999
Short course (≤2 years), *n* (%)	4 (36.3)	2 (16.6)	0.3707	1 (11.1)	5 (35.7)	0.3401
Total MFD, no./mm^2^, mean (SD)	2070.2 (2337.5)	1141.7 (1154.1)	0.6891	1244.1 (1248.4)	1790.48 (2126.3)	0.9669
Small MFD, no./mm^2^, mean (SD)	1296.3(1430.8)	898.5 (973.6)	0.8064	983.0 (1012.9)	1168.9 (1310.6)	0.7505
Large MFD, no./mm^2^	773.9 (930.1)	227.6 (264.6)	0.0371*	261.1 (294.5)	621.7 (849.7)	0.5648
Small MF/Large MF ratio, mean (SD)	1.9 (0.9)	5.9 (4.5)	<0.0001***	5.9 (5.6)	3.1 (2.3)	0.0671

Abbreviations: AN, autonomic neuropathy; IQR, interquartile range; MF, myelinated nerve fibers; MFD, myelinated nerve fibers density; n, numbers; NA, not available; SD, standard deviation; SMPN, sensory‐motor polyneuropathy; y, years;

A 2‐year course was considered the boundary between long and short courses.

Large MFD and small MF/large MF ratio were not available in five cases for severely loss of large MFD (large MFD = 0/mm^2^).

*, **, ***Significant difference at 0.05, 0.01, 0.001 levels, respectively.

#### *Early‐onset versus late‐onset* *ATTR‐PN*

There was no significant difference in disease durations, and the small and total MFD were similar between early‐onset and late‐onset cases. However, the late‐onset ATTR‐PN patients had more severe loss of large MF and higher small/large MF ratios (all *p* < 0.05). (Table 2).

#### *Val30 Met* *versus* *non‐ Val30Met ATTR‐PN*

There was no significant difference in disease durations or in large, small, and total MFD between patients with Val30Met mutation and non‐Val30Met mutation (all *p* <0.05). But patients in the Val30Met group had older age of onset and a higher proportion of positive family history (Table 2).

#### *Duration of disease and* *MFD*

**Figure 3:**
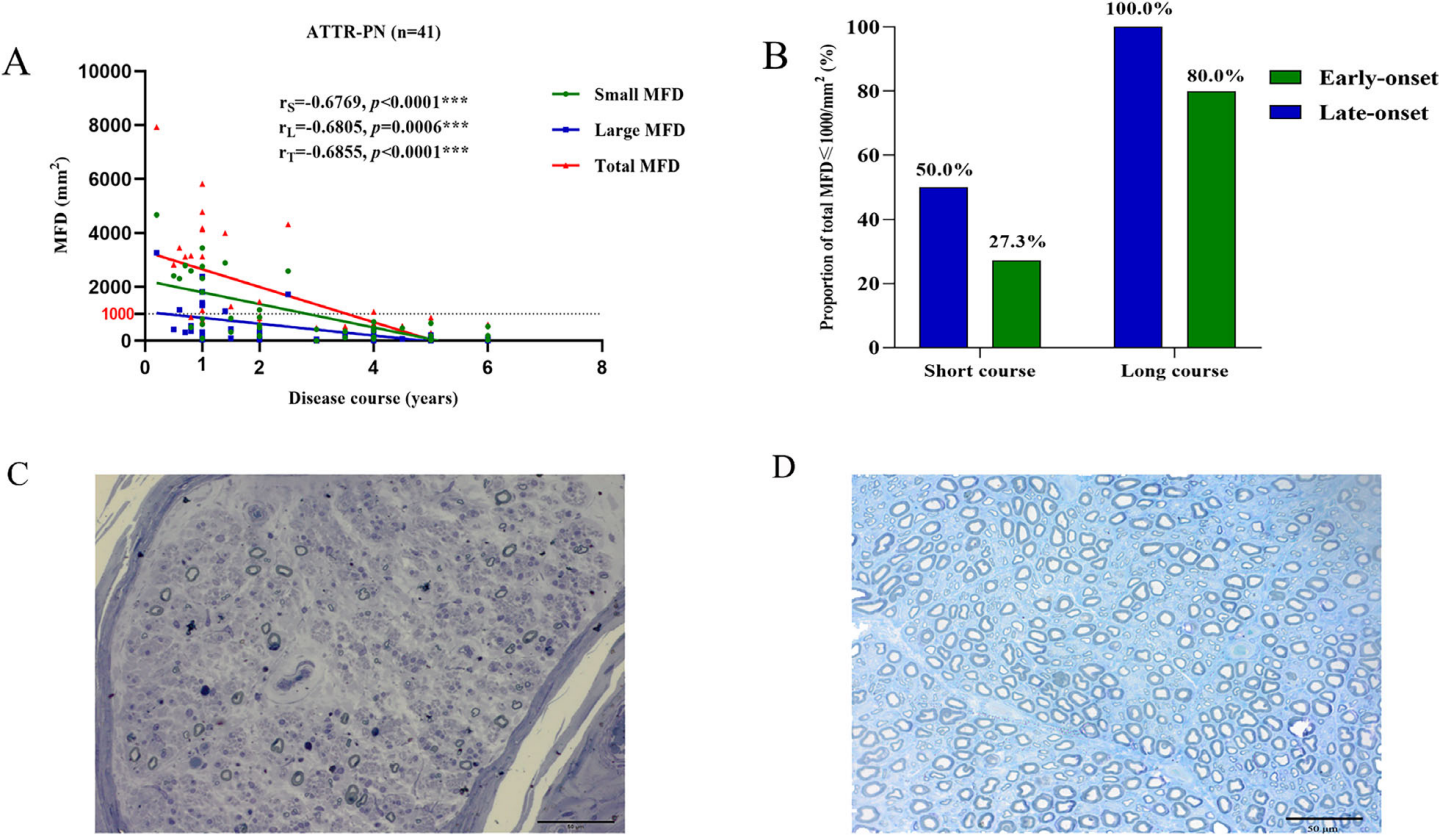
Correlations between disease course and MFD of ATTR‐PN. The longer the disease course, the more severe MFD loss could be observed in small, large, and total MFD (A). Both early‐ and late‐onset patients had a low proportion of total MFD ≤ 1000/mm^2^ in a short disease course compared with that of a long course. However, loss of total MFD of all late‐onset patients was severe (B). (C) and (D) showed a patient with 1100/mm^2^ of total MFD, and a normal control with 10300/mm^2^. ***Significant correlation at 0.001 level.

The small, large, and total MFD were negatively correlated to the disease courses in ATTR‐PN patients (r = 0.6769, *p* < 0.0001; r = 0.6805, *p* = 0.0006; r = 0.6855, *p* < 0.0001, respectively). Furthermore, the proportion of cases, whose total MFD were lower than 1000/mm^2^, was higher in the long disease course group than in the short disease course group, whether the cases were early‐onset (*p* = 0.0300) or late‐onset (*p* = 0.0409). However, MFD were lower than 1000/mm^2^ among more late‐onset cases than early‐onset ones, especially in the early disease stage (50.0% vs. 27.3%), but without statistical difference (*p* > 0.05). For the long course group (longer than 2 years), all the late‐onset patients and most of the early‐onset patients had severe MFD reductions, which were lower than 1000/mm^2^ (Fig. 3).

#### *Correlation analysis between* *MFD* *and clinical data of* *ATTR‐PN*

For clinical measurement scales, negative correlations between MFD and NIS, Norfolk QOL‐DN score were observed with significant difference (*p* < 0.05), as well as negative correlations between NIS, Norfolk QOL‐DN, and duration (*p* < 0.05). Correlations between COMPASS‐31 and MFD, duration of disease were not observed in this study (*p* > 0.05) (Table 3).

**Table 3:** Correlation analysis between MFD and clinical data of ATTR‐PN patients.

	Small MFD	Large MFD	Total MFD	Duration of disease
NIS	r = −0.5835, *p* = 0.0055**	r = −0.7147, *p* = 0.0003***	r = −0.6790, *p* = 0.0007***	r = 0.5278, *p* = 0.0067**
Norfolk QOL‐DN	r = −0.4842, *p* = 0.0305*	r = −0.6466, *p* = 0.0021**	r = −0.5880, *p* = 0.0064**	r = 0.5195, *p* = 0.0093**
COMPASS‐31	r = −0.1792, *p* = 0.4370	r = −0.2714, *p* = 0.2340	r = −0.1792, *p* = 0.4370	r = 0.2746, *p* = 0.1840

Abbreviations: COMPASS‐31, Composite Autonomic Symptom Score31; MFD, myelinated nerve fibers density; NIS, Neuropathy Impairment Score; Norfolk QOL‐DN, Norfolk Quality of life‐diabetic neuropathy score.

*, **, ***Significant correlation at 0.05, 0.01, 0.001 levels, respectively.

The data were available for correlation analysis only in 21 values of NIS, 21 values of COMPASS‐31, and 20 values of Norfolk QOL‐DN, respectively.

All significant *p* values are printed in bold.

## Discussion

The most common misdiagnosed disease of ATTR‐PN is CIDP, due to the similar sensory‐motor symptoms, decreased tendon reflex, electrophysiological demyelination, and cerebrospinal fluid cytoalbuminologic dissociation.^6^ Pathologically, apart from demyelinating features like thin myelin sheath and atypical onion balls, CIDP patients also presented with axonal involvements occasionally.^21^ The MFD could usually provide some other identifiable clues. It was found in our study that the MFD reduction in case of CIDP was milder than that of ATTR‐PN, and the extent of MFD reduction per year might be a helping indicator when confusing with the two diseases according to our results. Our observations were consistent with Ikeda S et al's study. In case of typical CIDP, the mean total MFD was 6060/mm^2^, with a disease duration of 18.7 months.^21^ CIAP patients usually had a longer disease duration than those with ATTR‐PN, but with milder MFD reduction. The mean total MFD was 3253/mm^2^ with an average disease duration of 41 months in Vrancken AF et al's study.^12^Our study proved that the reduction of total MFD of ATTR‐PN patients were more prominent than those of CIAP, especially for small MFD. Clinically, CIAP usually presents with slowly progressive sensory or sensory‐motor polyneuropathy, accompanied by mild or no autonomic dysfunction.^13^, ^22^ However, patients with ATTR‐PN manifested obvious autonomic dysfunction and a progressive duration. If left untreated, the patients usually had no more than 7–10 years of survival.^10^ Thus, the disease duration dependent reduction of MFD could help distinguish ATTR‐PN from CIAP with a high sensitivity and specificity. Compared with ATTR‐PN, the MFD reduction in LO‐CMT2 cases had distinct characteristics with more predominant reduction of large MFD. Conversely, small MFD was relatively reserved. Furthermore, autonomic dysfunction was less common, even in case of a long disease duration.^23^ Pathologically, the sural nerve biopsy of CMT2 patients revealed loss of large MF with regeneration clusters.^24^ Both POEMS syndrome and ATTR‐PN could present with electrophysiological features of both demyelinating and axonal impairment, and the axonal degeneration of the two was more severe than that of CIDP,^2^, ^25^ which could also be observed pathologically.^4^, ^26^ Apart from neovascularization of epineurial vessels,^26^ MFD might give us some hints of this disease with relatively moderate loss of both large and small MF as we observed. Vasculitic neuropathy clinically presented with an acute/subacute, relapsing, multifocal neuropathy, which was a diverse group of diseases with vascular damage and ischemic injury histopathological and diagnosed only by pathology.^27^ In addition, the MFD of sural nerves usually significant reduced inhomogenously.^12^, ^28^ We proved that the severe loss of MF occurred in both ATTR‐PN and vasculitic neuropathy. Furthermore, the loss was more rapid in a disease duration dependent manner in vasculitic neuropathy. Toxic neuropathy usually presented with loss of both small and large MF, yet the severity of MFD reduction varied according to toxins.^29^, ^30^ So, the history of exposure to toxins was vital. However, if the toxins were hypotoxic, prominent reduction of the MFD might not be observed, especially when toxin exposure was discontinued.

In general, both ATTR‐PN patients and vasculitic neuropathy patients had the most severe MF loss, and the former was relatively insidious. Interestingly, our study also proved that only in the ATTR‐PN group was the MFD negatively correlated with the course of disease, indicating ATTR‐PN was a relatively severe chronic progressive disease that was similar to neurodegenerative disorders, such as Alzheimer's disease, Parkinson's disease, and Amyotrophic lateral sclerosis, with abnormal protein aggregation and no relapse or remission in the course of disease.^31^ Moreover, MF loss in the asymptomatic stage had been reported. That was, pathological changes preceded clinical symptoms by several years,^20^ which often occurred in degenerative diseases. The neurodegenerative disease‐like feature of ATTR‐PN might be critical to differential diagnosis with other common peripheral neuropathies. A good knowledge of the characteristics of ATTR‐PN could contribute much to correct diagnosis.

Previous studies revealed that the longer the course of disease was, the higher positive propotion the nerve Congo red staining had. There were mild or no amyloid deposits in the early disease stage despite the severe loss of MF.^3^ Similar results were observed in our study. Amyloid deposits were not easily observed in cases with a short disease duration, especially in late‐onset cases, which might increase the chance of misdiagnosis of ATTR‐PN cases in the early disease stage. Furthermore, the form of amyloid fibrils in late‐onset patients was usually pattern A, which was thin and short, with less congophilic and apple‐green birefringence under polarized light, which might partly account for the low positive rate of Congo red staining in late‐onset patients. Pattern A amyloid deposits were mainly found in late‐onset patients in Sweden and non‐endemic areas of Japan.^32^, ^33^, ^34^

In our study, large MF was impaired more severely in late‐onset patients than in early‐onset ones. In another ATTR‐PN study of Val30Met mutation, sural nerve biopsy specimens showed predominantly small‐fiber loss in early‐onset cases.^3^ In the dorsal root ganglia, amyloid deposits and neuronal cell loss were conspicuous in early‐onset cases, while these were less severe in late‐onset cases. The mean diameter of remaining dorsal root ganglion neurons was larger in early‐onset cases as compared with normal controls, suggesting predominant loss of small neurons.^3^ Furthermore, electron microscopic observation has revealed that the direct damage to Schwann cells by amyloid fibrils might be the mechanism underlying the loss of small MF in ATTR‐PN, especially in early‐onset patients.^35^ In late‐onset cases, blood–nerve barrier disruption might also participate in the pathogenesis of neuropathy, regardless of the presence or absence of amyloid deposition,^35^ which might cause more loss of large MF and rapid progression. We found that in some of the ATTR‐PN patients in the early stage (within 2 years), MFD had been dramatically reduced, especially in late‐onset cases. Furthermore, in the course of more than 2 years, MFD of all late‐onset cases were below the limit of 1000/mm^2^, offering more evidence that ATTR‐PN was a malignant and progressive disease. So, for late‐onset patients with polyneuropathies of more than 2 years disease courses, the diagnosis of ATTR‐PN should be cautious, if no severe MFD reduction have not been observed. The pathological findings can also help improve the efficiency of *TTR* gene detection.

In this study, we found that the MFD were positively correlated with the NIS, Norfolk QOL‐DN scores of ATTR‐PN, which was more pronounced in large MFD, but there was no significant correlation between MFD and the COMPASS 31. These findings suggested that the loss of sensory MF morphologically, especially large sensory MF, was consistent with the severity of neuropathy and functional impairment in ATTR‐PN patients. However, small MF, not including unmyelinated nerve fibers, could not reflect autonomic nerve impairment, which was why COMPASS 31 was not correlated with the small MFD.

This study had several limitations. First, the sample size was relatively small. Second, classification of subtypes was lacking in disease groups, such as CIDP, vasculitic neuropathy, and toxic neuropathy groups, which made it difficult to gain keen insights into the disease duration‐dependent loss of MF.

In conclusion, the MFD observation of nerve tissues is useful for differentiation between ATTR‐PN and other common peripheral neuropathies, especially in Congo red negative cases. It was found that the loss of the sural nerve fibers density in patients with ATTR‐PN was significantly related to the course of peripheral neuropathy, and the loss of nerve fibers was more serious than in most of other common peripheral neuropathies. The pattern of large and small MF loss is different in early‐onset and late‐onset cases. Loss of sensory nerve fibers is consistent with the severity of neuropathy and functional scores.

## Author Contributions

KD: acquisition of data, completion of statistical analysis, and drafting of the initial manuscript and writing of the final manuscript. XC: acquisition of data, study concept and design, and critical revision of the manuscript. YT, MY, YZ, JD, HL, WZ, and ZW: study concept and design, and critical revision of the manuscript. YY and LM: data review, interpretation of results, and revision of the initial draft.

## Conflict of Interest

The authors declare no conflict of interest.

## Data Availability

Anonymized data will be shared by request from any qualified investigator.

## References

[1] Tozza S , Severi D , Spina E , et al. The neuropathy in hereditary transthyretin amyloidosis: a narrative review. J Peripher Nerv Syst. 2021;26:155‐159. 33960565 10.1111/jns.12451 PMC8360044

[2] Lozeron P , Mariani LL , Dodet P , et al. Transthyretin amyloid polyneuropathies mimicking a demyelinating polyneuropathy. Neurology. 2018;91:e143‐e152. 29907605 10.1212/WNL.0000000000005777

[3] Koike H , Misu K , Sugiura M , et al. Pathology of early‐ vs late‐onset TTR Met30 familial amyloid polyneuropathy. Neurology. 2004;63:129‐138. 15249622 10.1212/01.wnl.0000132966.36437.12

[4] Luigetti M , Romozzi M , Bisogni G , et al. hATTR pathology: nerve biopsy results from Italian referral centers. Brain Sci. 2020;10:780. 10.3390/brainsci10110780 PMC7692609 33114611

[5] Cakar A , Durmus‐Tekce H , Parman Y . Familial amyloid polyneuropathy. Noro Psikiyatr Ars. 2019;56:150‐156. 31223250 10.29399/npa.23502 PMC6563867

[6] Cortese A , Vegezzi E , Lozza A , et al. Diagnostic challenges in hereditary transthyretin amyloidosis with polyneuropathy: avoiding misdiagnosis of a treatable hereditary neuropathy. J Neurol Neurosurg Psychiatry. 2017;88:457‐458. 28188196 10.1136/jnnp-2016-315262 PMC5529976

[7] Du K , Li F , Wang H , et al. Hereditary transthyretin amyloidosis in mainland China: a unicentric retrospective study. Ann Clin Transl Neurol. 2021;8:831‐841. 33739616 10.1002/acn3.51328 PMC8045954

[8] Adams D , Cauquil C , Labeyrie C . Familial amyloid polyneuropathy. Curr Opin Neurol. 2017;30:481‐489. 28678039 10.1097/WCO.0000000000000476

[9] Luigetti M , Conte A , Del GA , et al. TTR‐related amyloid neuropathy: clinical, electrophysiological and pathological findings in 15 unrelated patients. Neurol Sci. 2013;34:1057‐1063. 22592564 10.1007/s10072-012-1105-y

[10] Adams D , Ando Y , Beirao JM , et al. Expert consensus recommendations to improve diagnosis of ATTR amyloidosis with polyneuropathy. J Neurol. 2021;268:2109‐2122. 31907599 10.1007/s00415-019-09688-0 PMC8179912

[11] European Federation of Neurological Societies/Peripheral Nerve Society Guideline on management of paraproteinemic demyelinating neuropathies. Report of a Joint Task Force of the European Federation of Neurological Societies and the Peripheral Nerve Society‐‐first revision. J Peripher Nerv Syst. 2010;15:185‐195. 21040140 10.1111/j.1529-8027.2010.00278.x

[12] Vrancken AF , Notermans NC , Jansen GH , Wokke JH , Said G . Progressive idiopathic axonal neuropathy–a comparative clinical and histopathological study with vasculitic neuropathy. J Neurol. 2004;251:269‐278. 15015005 10.1007/s00415-004-0275-9

[13] Notermans NC , Wokke JH , van der Graaf Y , Franssen H , van Dijk GW , Jennekens FG . Chronic idiopathic axonal polyneuropathy: a five year follow up. J Neurol Neurosurg Psychiatry. 1994;57:1525‐1527. 7798984 10.1136/jnnp.57.12.1525 PMC1073236

[14] Richards S , Aziz N , Bale S , et al. Standards and guidelines for the interpretation of sequence variants: a joint consensus recommendation of the American College of Medical Genetics and Genomics and the Association for Molecular Pathology. Genet Med. 2015;17:405‐424. 25741868 10.1038/gim.2015.30 PMC4544753

[15] Dispenzieri A . POEMS syndrome: 2019 update on diagnosis, risk‐stratification, and management. Am J Hematol. 2019;94:812‐827. 31012139 10.1002/ajh.25495

[16] Collins MP , Dyck PJ , Gronseth GS , et al. Peripheral nerve society guideline on the classification, diagnosis, investigation, and immunosuppressive therapy of non‐systemic vasculitic neuropathy: executive summary. J Peripher Nerv Syst. 2010;15:176‐184. 21040139 10.1111/j.1529-8027.2010.00281.x

[17] Staff NP , Grisold A , Grisold W , Windebank AJ . Chemotherapy‐induced peripheral neuropathy: a current review. Ann Neurol. 2017;81:772‐781. 28486769 10.1002/ana.24951 PMC5656281

[18] Xu CW , Wu HF , Chen J . Clinical and electrophysiological features and treatment of acrylamide‐induced toxic peripheral neuropathy. Zhonghua Lao Dong Wei Sheng Zhi Ye Bing Za Zhi. 2020;38:45‐47. 32062896 10.3760/cma.j.issn.1001-9391.2020.01.010

[19] Du K , Meng L , Lv H , et al. Sural biopsy to detect the axonal cytoskeleton defects in KIF5A‐related Charcot‐Marie‐tooth disease type 2. Clin Neuropathol. 2020;40:142‐149. 10.5414/NP301323 33155544

[20] Fernandes A , Coelho T , Rodrigues A , et al. Clinicopathological correlations of sural nerve biopsies in TTR Val30Met familial amyloid polyneuropathy. Brain Communications. 2019;1:fcz032. 32954271 10.1093/braincomms/fcz032 PMC7425381

[21] Ikeda S , Koike H , Nishi R , et al. Clinicopathological characteristics of subtypes of chronic inflammatory demyelinating polyradiculoneuropathy. J Neurol Neurosurg Psychiatry. 2019;90:988‐996. 31227562 10.1136/jnnp-2019-320741

[22] Teunissen LL , Notermans NC , Franssen H , et al. Differences between hereditary motor and sensory neuropathy type 2 and chronic idiopathic axonal neuropathy. A Clinical and Electrophysiological Study. Brain. 1997;120(Pt 6):955‐962. 9217680 10.1093/brain/120.6.955

[23] Pareyson D , Scaioli V , Laura M . Clinical and electrophysiological aspects of Charcot‐Marie‐tooth disease. Neuromolecular Med. 2006;8:3‐22. 16775364 10.1385/nmm:8:1-2:3

[24] Fu J , Dai S , Lu Y , et al. Similar clinical, pathological, and genetic features in Chinese patients with autosomal recessive and dominant Charcot‐Marie‐tooth disease type 2K. Neuromuscul Disord. 2017;27:760‐765. 28495047 10.1016/j.nmd.2017.04.001

[25] Ohashi N , Kodaira M , Morita H , Sekijima Y . Electrophysiological demyelinating features in hereditary ATTR amyloidosis. Amyloid. 2019;26:15‐23. 10.1080/13506129.2018.1564903 30688105

[26] Piccione EA , Engelstad J , Dyck PJ , Mauermann ML , Dispenzieri A , Dyck PJB . Nerve pathologic features differentiate POEMS syndrome from CIDP. Acta Neuropathol Commun. 2016;4:116. 27799073 10.1186/s40478-016-0389-1 PMC5088652

[27] Collins MP , Hadden RD . The nonsystemic vasculitic neuropathies. Nat Rev Neurol. 2017;13:302‐316. 28447661 10.1038/nrneurol.2017.42

[28] Zhang Y , Wan Y , Zhang B , et al. Clinico‐electrophysiological and pathological characteristics of neuropathy in Sjogren's syndrome: a report of 5 cases. Zhonghua Yi Xue Za Zhi. 2014;94:855‐858. 24854756

[29] Qiao X , Li Y , Hong D , et al. Clinical and pathological features of organophosphate induced delayed polyneuropathy. J Brain Nerv Dis. 2010;18:290‐293.

[30] Wang Z , Zhang J , Shi X , et al. Two cases of peripheral nerve and blood vessel damage caused by acrylamide poisoning. Chin J Prev Med. 2005;39:68.

[31] Dugger BN , Dickson DW . Pathology of neurodegenerative diseases. Cold Spring Harb Perspect Biol. 2017;9:a028035. 28062563 10.1101/cshperspect.a028035 PMC5495060

[32] Koike H , Nishi R , Ikeda S , et al. The morphology of amyloid fibrils and their impact on tissue damage in hereditary transthyretin amyloidosis: an ultrastructural study. J Neurol Sci. 2018;394:99‐106. 30243104 10.1016/j.jns.2018.09.011

[33] Bergstrom J , Gustavsson A , Hellman U , et al. Amyloid deposits in transthyretin‐derived amyloidosis: cleaved transthyretin is associated with distinct amyloid morphology. J Pathol. 2005;206:224‐232. 15810051 10.1002/path.1759

[34] Ihse E , Rapezzi C , Merlini G , et al. Amyloid fibrils containing fragmented ATTR may be the standard fibril composition in ATTR amyloidosis. Amyloid. 2013;20:142‐150. 23713495 10.3109/13506129.2013.797890

[35] Koike H , Ikeda S , Takahashi M , et al. Schwann cell and endothelial cell damage in transthyretin familial amyloid polyneuropathy. Neurology. 2016;87:2220‐2229. 27794111 10.1212/WNL.0000000000003362

